# Delayed Pneumoperitoneum After Percutaneous Endoscopic Gastrostomy (PEG) Tube Placement: A Case Report

**DOI:** 10.7759/cureus.57134

**Published:** 2024-03-28

**Authors:** Vaidehi Patel, Robert J Dabek, Fawaz Araim, Shirali Patel, Thomas J Kang

**Affiliations:** 1 General Surgery, Ascension Saint Agnes Hospital, Baltimore, USA

**Keywords:** intraperitoneal free air, feeding tube, peg, pneumoperitoneum, peg complication

## Abstract

When used for a selected patient population, percutaneous endoscopic gastrostomy (PEG) can provide enteral nutrition percutaneous endoscopic gastrostomy (PEG) safely. PEG tubes generally possess a very low chance of life-threatening complications but due to the patient population that requires PEG tubes, a delayed diagnosis of minor complications could be fatal. In this study, we present a case of delayed pneumoperitoneum, discovered weeks after our patient underwent PEG placement for enteral nutritional needs. The patient recovered without the need for operative intervention. The development of a pneumoperitoneum in the setting of recent PEG needs a thorough clinical evaluation, and caution must be taken before immediately proceeding to operative exploration.

## Introduction

A percutaneous endoscopic gastrostomy (PEG) tube serves as a favorable means of feeding and providing nutrition in patients with a functional GI system who require long-term enteral nutrition, typically for more than four weeks. Three modes can be used to place a gastrostomy tube: fluoroscopy-guided gastrostomy, percutaneous endoscopic gastrostomy, or surgical (laparoscopic or open) gastrostomy. After comparing several studies, the percutaneous gastrostomy tube method was found to be the most effective due to significantly lower rates of inpatient adverse events, mortality, and readmission rates compared with fluoroscopy-guided gastrostomy and surgical gastrostomy [1]. Although it’s an effective method of feeding with less side effects, increased usage of PEG tubes has resulted in more frequent occurrence of complications such as bleeding, wound infections, inadvertent tube removal, pneumoperitoneum, tube blockage, bowel perforation, and buried bumper syndrome [2]. High clinical standards of care and early screening methods are essential to prevent these complications and the morbidity and mortality associated with each. Included in this study, we present a case of PEG tube-related delayed pneumoperitoneum in a patient with a medical history of a cerebrovascular accident requiring PEG placement.

## Case presentation

A 66-year-old African American male presented to the emergency department from a skilled nursing facility with complaints of abdominal pain. The patient had a cerebrovascular accident two months prior, requiring extended hospitalization and eventual percutaneous endoscopic gastrostomy (PEG) tube placement 5 weeks prior due to persistent weakness and dysphagia. The patient exhibited persistent right-sided weakness and dysarthria; therefore, the majority of the history was obtained from medical chart review, facility caretakers, and family members. Of note, other comorbid conditions included hypertension and obesity with prior laparoscopic sleeve gastrectomy. Per facility caretakers, he had complained of pain in the abdomen, particularly at the PEG tube site for the last two to three 2-3 days. The pain progressed, culminating in his refusal to allow any manipulation of the tube, including administration of tube feedings and routine free water flushes. At this time there was increased purulent-appearing drainage seen at the PEG insertion site, which ultimately prompted the emergency room visit. The facility did not note any abnormalities in his vital signs, nor did they report any other acute changes prior to his arrival.

**Figure 1:**
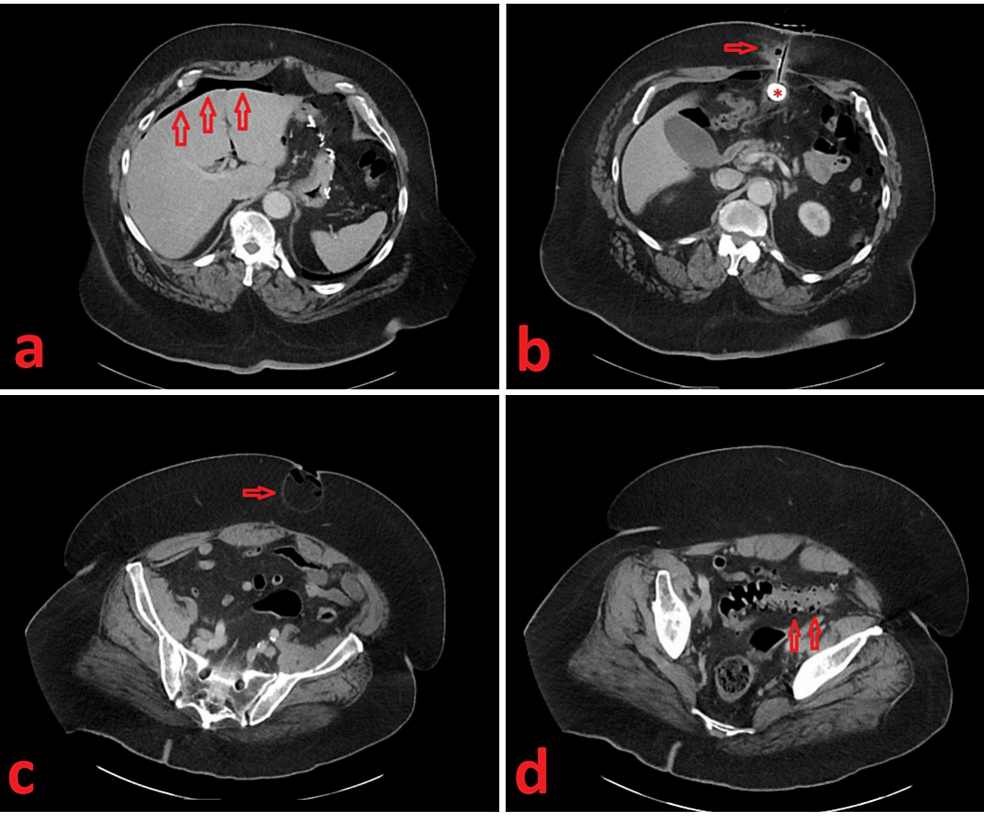
Contrast-enhanced computed tomography (CT) scan of the abdomen and pelvis Axial images of an intravenous contrast-enhanced computed tomography (CT) scan of the abdomen and pelvis demonstrating (a) pneumoperitoneum (arrows), (b) abscess within the subcutaneous tissue along the PEG tract (arrow), and appropriate positioning of the PEG balloon within the gastric sleeve (*), (c) an umbilical hernia containing fat with air and minimal overlying skin with concern for ulceration (arrow), and (d) extensive colonic diverticulosis (arrows).

Upon arrival at the emergency department, vital signs were within normal limits, and physical examination by an emergency physician was notable for abdominal tenderness and purulent discharge from the PEG site. Initial laboratory studies, including complete blood count, basic metabolic panel, and lactic acid did not reveal any abnormalities. His white blood count (WBC) was 4,000 /µL. A contrast-enhanced computed tomography (CT) scan of the abdomen and pelvis was obtained. Pertinent findings (Figure 1) included pneumoperitoneum, abscess at the PEG tube site within the subcutaneous tissues, gastrostomy tube position with inflated balloon within the gastric sleeve, extensive colonic diverticulosis without clearly identifiable inflammatory changes, and an umbilical hernia containing intra-abdominal fat and air with questionable erosion through the overlying skin. Surgical consultation was sought at this time.

**Figure 2:**
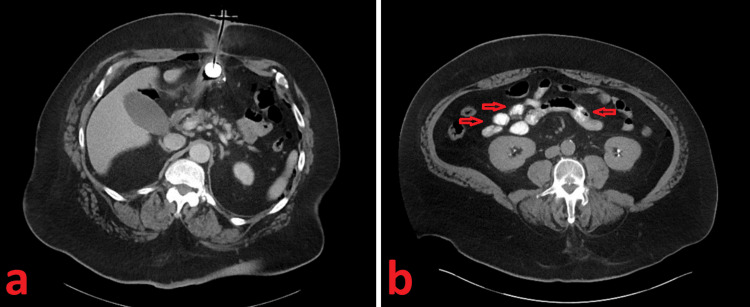
Computed tomography (CT) scan of the abdomen and pelvis with oral contrast Axial images of oral contrast-enhanced computed tomography of the abdomen and pelvis demonstrating (A) appropriate positioning of the PEG tube without evidence of extravasation of contrast and (B) contrast within loops of the small bowel, again without any evidence of extravasation.

Physical exam findings were significant for regional tenderness and purulent drainage at the PEG site without any apparent tenderness in the lower quadrants or periumbilical areas. There were no skin changes or drainage noted at the site of the umbilical hernia. A decision was made to obtain additional cross-sectional imaging with the addition of oral contrast administration through the PEG tube to confirm appropriate positioning and rule out leaks or perforation. CT findings did not show any extraluminal contrast extravasation with contrast advancing to the small intestines (Figure 2).

The patient was admitted for observation and treated with IV antibiotics and local wound care. It was felt that the abscess was actively draining and, as such, no drainage procedure was performed. He was evaluated by a speech pathologist on hospital day one and was recommended for a soft diet with thin liquids. The patient was able to tolerate 50% of his meals without any problems. There was no further imaging acquired as the abdomen remained soft, non-tender, and non-distended. The gastrostomy tube was able to be used for medications as needed. Recommendations were made by the surgery team to remove the PEG tube once the patient demonstrated adequate PO (per os) caloric intake. He was discharged back to his nursing home facility on hospital day four with a seven-day course of PO Bactrim.

## Discussion

The number of patients requiring PEG tubes has increased in the last decade and will continue to increase as more patients present with comorbidities that disable them from acquiring the required nutrition to sustain proper body function. Early pneumoperitoneum is common after PEG procedures, with an incidence of up to 50% [3]. Since there is high intragastric air pressure during endoscopy, air may escape during needle puncture and the passage of the PEG tube through the abdominal wall, resulting in free intra-abdominal air. Blum et al. retrospectively reviewed 722 cases of patients who underwent PEG/PEG-J (percutaneous endoscopic gastro-jejunostomy) at their institute. Of 722 patients, 320 (44 %) had abdominal radiographs or abdominal CT imaging within five days. Thirty-nine (5%) patients were found to have free air after PEG/PEG-J placement. Out of those 39, 33 (85%) had “benign pneumoperitoneum” and were discharged without complication or surgical intervention. Of the six patients with serious complications related to their procedure, five (83%) had clinical signs of intra-abdominal complications (peritonitis) that helped guide their management [4]. The authors proposed an algorithm for the management of early findings of pneumoperitoneum. They suggested that patients with concomitant free fluid, or those exhibiting peritonitis, required emergent surgical intervention.

The above studies suggest that transient clinical pneumoperitoneum is a common finding post-PEG tube placement, which could be solved with conservative treatment if there are no peritoneal signs or free fluid present. A recent randomized trial showed that patients who had insufflation with carbon dioxide had more rapid reabsorption and resolution of their pneumoperitoneum than those who received ambient air [5]. The pneumoperitoneum is usually self-limiting and does not persist for more than 72 hours. Persistence of free air after 72 hours is considered a complicated pneumoperitoneum requiring intervention to prevent further damage.

The patient population that qualifies for PEG placement has severe morbidity. Given these findings, clinically relevant pneumoperitoneum after PEG insertion is likely to have significant signs such as fever, abdominal tenderness, or leukocytosis. However, most patients are asymptomatic. Moreover, some patients undergoing PEG tube insertion may receive broad-spectrum antibiotics because of co-existent infection (e.g., aspiration pneumonia and urinary tract infection). In these patients, clinically significant signs may be masked, and complicated pneumoperitoneum could be missed. Furthermore, peritoneal signs might be overlooked due to altered mental status (patients after cranial neurosurgery, dementia, post-CVA), long-term sedation (patients receiving mechanical ventilation), or staff shortages (nursing home, rehabilitation facility) [6]. In fact, dysphagia post-cerebral vascular accident (CVA) is the most common indication for PEG placement [7]. If peritoneal signs develop, early intervention would be required, and if the clinical picture is obscured a workup, including abdominal CT, CBC, lactate, and a basic metabolic panel (BMP) may aid in diagnosis.

Our case is unique in that the pneumoperitoneum is discovered weeks after the PEG placement. The diagnosis is not immediately evident given the patient's inability to provide adequate history, and the physical exam was complicated by focal tenderness around the PEG site. Abdominal CT showed a moderate pneumoperitoneum with a small amount of intra-abdominal fluid. After repeat oral contrast abdominal CT confirmed the appropriate tube position without extravasation of contrast, we elected to proceed with nonoperative management. Our conservative treatment deviates from the previously suggested algorithm, which calls for operative intervention in the presence of free fluid. As such, it may be useful to separate early (within five days of procedure) and late post-PEG pneumoperitoneum (occurring after five days) as two separate entities. This appears logical as the mechanisms of pneumoperitoneum between the two entities differ. In our case, the air was likely introduced into the abdomen through the failure of complete fistulization of the gastrostomy tract due to localized infection.

## Conclusions

Pneumoperitoneum post-PEG tube insertion can occur as a late-onset finding. A CT scan is superior to X-rays in detecting the presence of a pneumoperitoneum. The development of a pneumoperitoneum needs a thorough clinical evaluation. Some of the difficulties that may arise are unique to the patient subset that requires PEG placement. The majority of the patients present with aphasia and/or dementia where physical exam findings and history may be unreliable. As such, caution must be taken before immediately proceeding with operative exploration for pneumoperitoneum in this subset of patients.
